# Editorial Ethics

**Published:** 1902-06

**Authors:** 


### EDITORIALS.

### EDITORIAL ETHICS.

In the May issue of the *American Veterinary Review* a report is given of the recent meeting of the Alumni Association of the New York American Veterinary College. It is stated that Dr. W. Horace Hoskins said, in response to a toast on "Journalism," that he hoped to see the medical periodicals eliminate the editorial page entirely, filling the journal with good original articles, and allowing the reader to draw his own conclusions. To this sentiment the *Review* reporter does not hesitate to take exception, for in the next sentence he makes clear his impression that such a publication would be too dry, that many advances are brought out in editorial discussion, etc.

Whatever one's opinion may be in regard to the retention or abolition of the editorial page, there can be no doubt that the prevailing sentiment of readers and the ethics of journalism are strongly opposed to mixing editorial utterances with news reports. One of the first things a reporter should learn is that he is to write reports or descriptions of things as they exist and not record his personal opinions.

It is clear that the *Review* is strongly in favor of editorials when the editor admits a report of a meeting in which, in effect, the reporter writes a dissenting editorial upon a remark made in response to a toast. That the editorial clause happened to be dissenting has no special bearing on the case. The question is, Is it a good plan for editors to state their personal opinions in juxtaposition with and evidently for the purpose of increasing or diminishing the effect of the remarks they are reporting? Is this not a perversion and abuse of the reportorial and editorial functions?

